# Reducing Disparities and Improving Mental Health for Transgender Persons

**DOI:** 10.31083/AP45278

**Published:** 2026-02-25

**Authors:** Osborne F.X. Almeida, Pedro Morgado

**Affiliations:** ^1^Max Planck Institute of Psychiatry, 80804 Munich, Germany; ^2^Life and Health Sciences Research Institute (ICVS), School of Medicine, University of Minho, 4710-057 Braga, Portugal

**Keywords:** gender identity, transgender health, minority stress, stigma and discrimination, gender-affirming care

## Abstract

This opinion piece primarily targets health and ancillary professionals, but it is also intended to serve as an unbiased guide for policymakers and legislators worldwide. It calls for the protection of human rights and advocates for evidence-based, inclusive health and socio-legal policies for all, regardless of gender identity. The article clarifies the distinctions between biological sex, sexual orientation, and gender, and critically examines and refutes the growing misconception that recognizing diverse gender identities is a political matter rather than a medical and human rights issue. In fact, gender diversity has existed across cultures for millennia. A major concern addressed in this piece is how societal norms expose transgender and gender non-conforming (TGNC) persons to stigma, discrimination, and social exclusion. The focus, however, is on the heightened vulnerability of TGNC individuals, particularly those from marginalized groups (for example, immigrants, individuals with low socio-economic status, non-White populations), to mental health conditions such as depression, anxiety, eating disorders, substance misuse, and suicidal ideation. Young TGNC persons and those with intersecting marginalized identities (for example, migratory background, socio-economic disadvantage, skin color) are especially susceptible to victimization. Many of these difficulties are exacerbated by discrimination, lack of legal protections, and societal prejudice. The article also addresses disparities in TGNC rights and protections across different regions, with the highest levels in Western Europe and North America and the lowest in the Middle East and Africa, and highlights how the scarcity of robust TGNC research in the Global South may adversely affect the health and well-being of TGNC individuals in those regions. The authors propose eight actionable recommendations to improve mental health outcomes for gender minorities: (i) collecting more nuanced data that distinguishesdifferentiates TGNC people from other sexsexual and gender minorities (under the Lesbian, Gay, Bisexual, Transgender, Queer + umbrella); (ii) fostering dialogue and research to counter misinformation about TGNC persons; (iii) incorporating gender diversity into early education; (iv) promoting human connectedness and social support; (v) developing inclusive mental healthcare services; (vi) improving professional training on gender diversity; (vii) establishing centres specializing in gender medicine; and (viii) banning harmful practices such as gender identity conversion efforts. The importance of cultural sensitivity when implementing these recommendations is emphasized.

## Main Points

1. Transgender and gender non-conforming (TGNC) people face a disproportionately high burden of mental health problems, including depression, anxiety, substance misuse, eating disorders, and suicidality, primarily as a consequence of stigma, discrimination, and minority stress rather than gender diversity itself.

2. Mental health outcomes for TGNC individuals are strongly shaped by structural and societal conditions, with worse outcomes in settings characterized by legal insecurity, social exclusion, and transphobic policies, particularly affecting young people and those with intersecting marginalized identities.

3. Reducing disparities in TGNC mental health requires coordinated, evidence-based action, including inclusive and gender-affirming healthcare, improved professional training, stronger legal protections, and the implementation of the eight actionable recommendations proposed by the authors.

## 1. A Global Perspective on Gender Identity: Recognition, Stigma, and Inequality

The narrative that acknowledgment of diverse gender identities is part of a political agenda rather than a recognition of human diversity, seems to be gaining currency just now. The “woke ideology” theory claims that the rise in the number of persons with identities outside the binary (heteronormative) gender expectation is due to “conditioning” and libertarian attitudes; this view is at odds with the reality that gender diversity has been historically recorded across on different continents [1, 2]. The popular flawed rhetoric runs counter to the Universal Declaration of Human Rights and calls out for better understanding and care of persons belonging to sexual or gender minorities.

Multidisciplinary research shows that sex and sexual orientation on the one hand, and gender and gender identity on the other, are distinct categories. Biological sex, which is assigned at birth on the basis of physical traits does not predict eventual sexual orientation which describes patterns of emotional, romantic, and sexual attraction to people of a particular gender (heterosexual, homosexual/lesbian, bisexual). In contrast, gender is a social construct that refers to social norms; like culture, ethnicity, and class, gender defines a person’s social identity [3].

Gender identity refers to a person’s own, inner, sense of self and gender (boy/man or girl/woman, both, or neither) which does not correspond to their birth-assigned sex. Gender realization peaks around 5 years of age but may continue through puberty and into adulthood [4]. Persons whose gender identity is incongruent with their natal sex are referred to as transgender and gender non-conforming (TGNC). Recent neuroimaging studies suggest that TGNC individuals have a unique brain phenotype in terms of structure and connectivity [5, 6]. Such biological differences between TGNC and heteronormative persons do not, however, imply that gender incongruence is a mental or behavioral disorder; in fact, transgenderism was de-pathologized in the WHO’s 11th Revision of its International Classification of Diseases (ICD-11) [7].

Given their overt gender expression/presentation, TGNC persons often become the victims of stigma, discrimination, and social exclusion, as well as verbal, emotional, and physical abuse—simply because their physical features, dress, accessories, grooming, behavior, voice, word choice, conversational mannerism etc are considered to deviate from traditional gender norms. Stereotypes shape perceptions of “otherness”, and may be exploited to marginalize “them”, i.e., those who are different from the majority [8, 9]. As a result, TGNC persons are often “edged towards the margins of society, where they get involved in risky situations and risky behaviours” [10], raising medical, social and legal issues and burdens.

We here focus on the mental health challenges of TGNC persons because of evidence that TGNC individuals are subject to more discrimination and violence than cisgender lesbian, gay or bisexual (LGB) and nonbinary (genderqueer, bigender, genderfluid) people^1^ (^1^Clarification of terminology: Lesbian, Gay, Bisexual, Transgender, Queer/Questioning, and others (LGBTQ+) is a broad term covering a spectrum of gender non-conforming persons. The term transgender is an umbrella term for people whose gender identity and/or expression is different from cultural expectations based on the sex they were assigned at birth. Being transgender does not imply any specific sexual orientation, i.e., transgender people may be straight, gay, lesbian, bisexual, etc. Non-binary is used to describe a person who does not identify exclusively as a man or a woman. Non-binary people may identify as being both a man and a woman (bigender), somewhere in between (gender fluid), or as falling completely outside these categories (agender, genderqueer). Although many non-binary persons also identify as transgender, not all non-binary people do. In this article, we use the term Transgender and Gender Non-Conforming (TGNC) an umbrella term to refer to individuals whose gender identities are incongruent with their assigned sex at birth. The term encompasses transgender men, transgender women, non-binary individuals, gender-fluid, and genderqueer individuals.). Proneness to discrimination and violence is greater in TGNC persons with disabilities and/or ethnic minority or migratory backgrounds. Across genders, trans women (male-to-female, MTF) and nonbinary people experience more violence than trans men (female-to-male, FTM) [11].

**Fig. 1.**
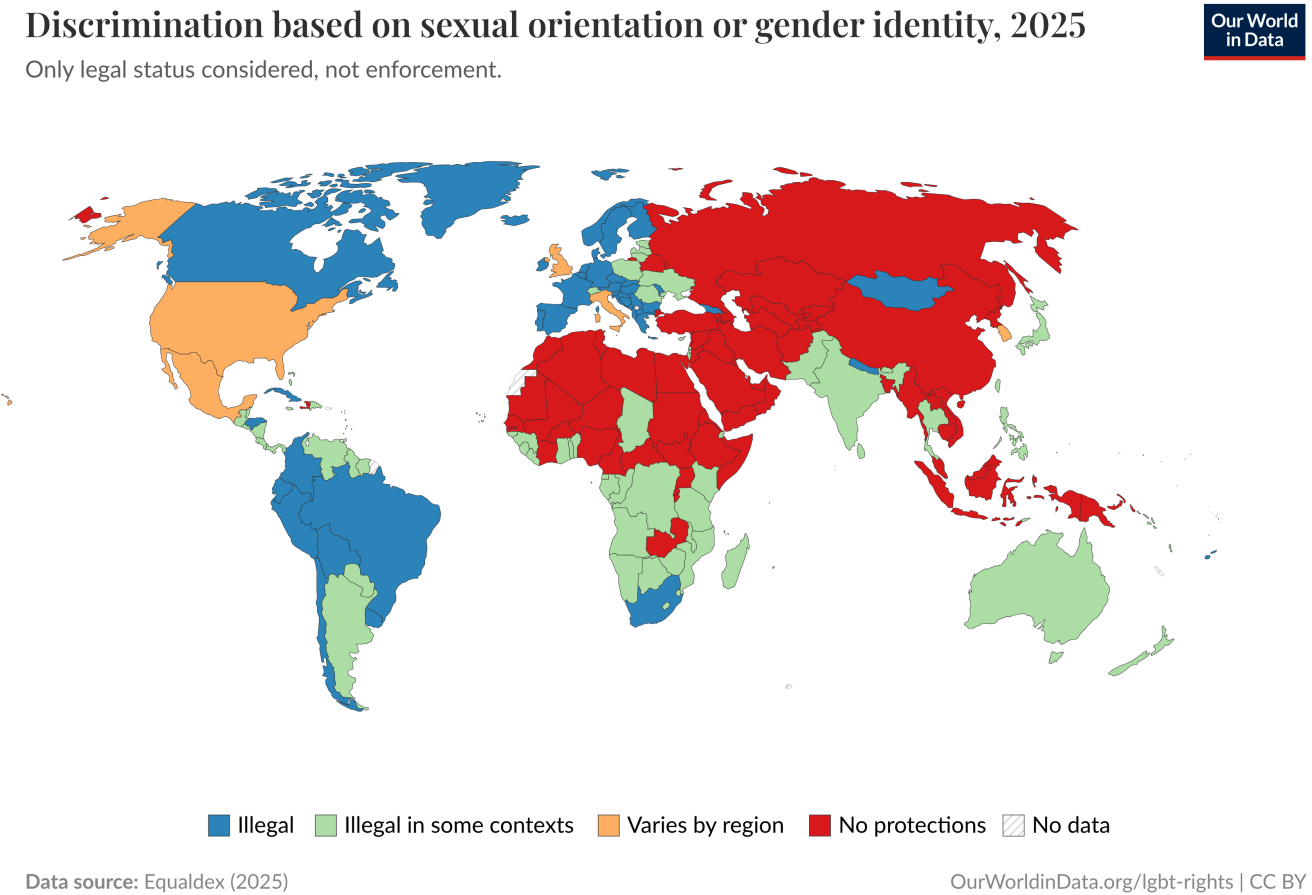
**Prohibition of discrimination based on sexual orientation and/or gender identity**. Data Page: “Discrimination based on sexual orientation or gender identity”, part of the following publication: Bastian Herre and Pablo Arriagada (2023)—“LGBT + Rights”. Data adapted from Equaldex. Retrieved from https://archive.ourworldindata.org/20250903-083611/grapher/discrimination-lgbt-equaldex.html [online resource] (archived on September 3, 2025). LGBT, Lesbian, Gay, Bisexual, Transgender.

Discrimination based on gender (and sexual orientation) remains partially or completely unregulated in large swathes of the world (Fig. 1). Besides their susceptibility to psychological distress and physical and verbal abuse, TGNC people are often deprived of medical and social services, as well as housing, educational and job opportunities, a situation further compounded by race and a range of socioeconomic factors [12]. Public stigma, often rooted in ignorance, along with structural stigma and discrimination driven by ideology, create stress for affected individuals and significantly worsen health and social inequalities [13, 14, 15]. This assertion is supported by two informative analyses of transgender rights: the Franklin & Marshall Global Barometer of Gay Rights (GBGR) which measured state and societal level human rights protection or persecution of TGNC people in 204 countries [16], and the Trans Rights Indicator Project (TRIP) which evaluated criminalization, legal recognition and legal protection of TGNC persons in 173 countries [17]. Both studies reported highly parallel patterns with respect to the rights of TGNC individuals—highest in Western Europe and North America, followed by (in rank order) countries in Eastern Europe and Central Asia, Latin America and the Caribbean, the Asian & Pacific Region, Sub-Saharan Africa and lastly, the Middle East and North African region. Increases in TRIP scores improved significantly in Eastern Europe and Central Asia, Latin America and the Caribbean, the Asian & Pacific Region in the 20 years up to 2020; in contrast scores in the Middle East and Africa remained consistently low in the same period. A careful analysis revealed that democratic regimes as well as higher levels of economic development tend to associate with greater TGNC rights whereas religion impedes progress in establishing equality for non-heteronormative persons [17].

**Fig. 2.**
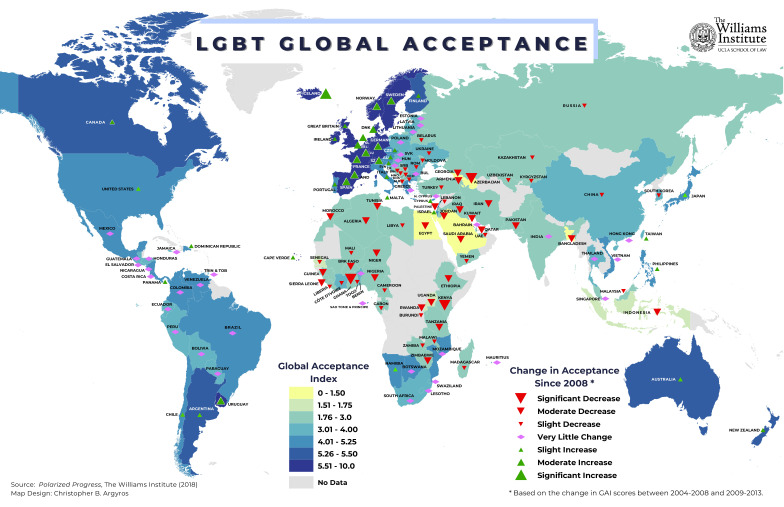
**Trends in acceptance of LGBT around the world**. (from The Williams Institute online infographics library https://williamsinstitute.law.ucla.edu/quick-facts/infographics/).

**Fig. 3.**
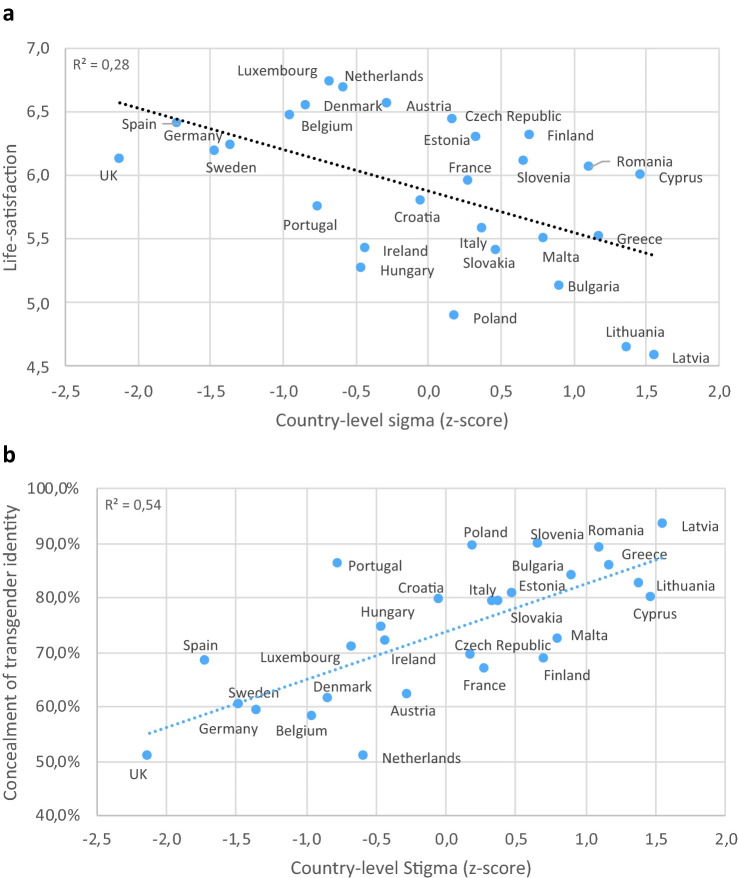
**Correlation of country-level stigma with life-satisfaction and identity concealment**. Mean country-level self-reported life-satisfaction (a) and mean proportion of transgender people reporting high level of concealment of their transgender identity (b) among transgender people across Europe by country-level structural stigma [20].

Importantly, many TGNC people suffer from the negative consequences of self-stigma (the internalization of public attitudes) [18]. While self-stigma, as well as the anticipation of discrimination, has long discouraged them from openly admitting their status when seeking help or treatment, newer generations (mostly in westernized cultures) are showing more openness about their TGNC status [19]. One interesting study in 28 European countries showed that, TGNC persons report greater “life satisfaction” in countries with low levels of gender identity-related stigma [20]. That same study found that concealment of transgender identity is lower in countries with low levels of gender-related stigma (Fig. 2). Unfortunately, these findings are likely to apply to only a few geo-politico-cultural regions since gender diversity is not universally accepted (Fig. 3, Ref. [20]), and there is data demonstrating a pronounced downward trend in its acceptability in certain regions (Eastern Europe, the Middle East/North Africa and Sub-Saharan Africa) since 1990 [21].

Published prevalence rates of transgenderism range from 0.1–3%. Assuming an average prevalence of 0.5% [10], the current global TGNC population is estimated at 30–40 million persons. This metric justifies concern not only from the perspective of human rights and economic output due to occupational dysfunction, but also health and well-being [22]. Some of the mental health issues faced by TGNC individuals are discussed briefly below.

Gender dysphoria, a state of clinically significant psychological distress and discomfort and listed in the Diagnostic and Statistical Manual of Mental Disorder (DSM), is common in the TGNC population [10, 23, 24]. The condition may become manifest during childhood and persist into adult life. Gender dysphoria may be partially related to the desire to undergo gender transitioning to align with the authentic self—a long, costly and complex process associated with social adjustments and legal formalities [25, 26].

**Fig. 4.**
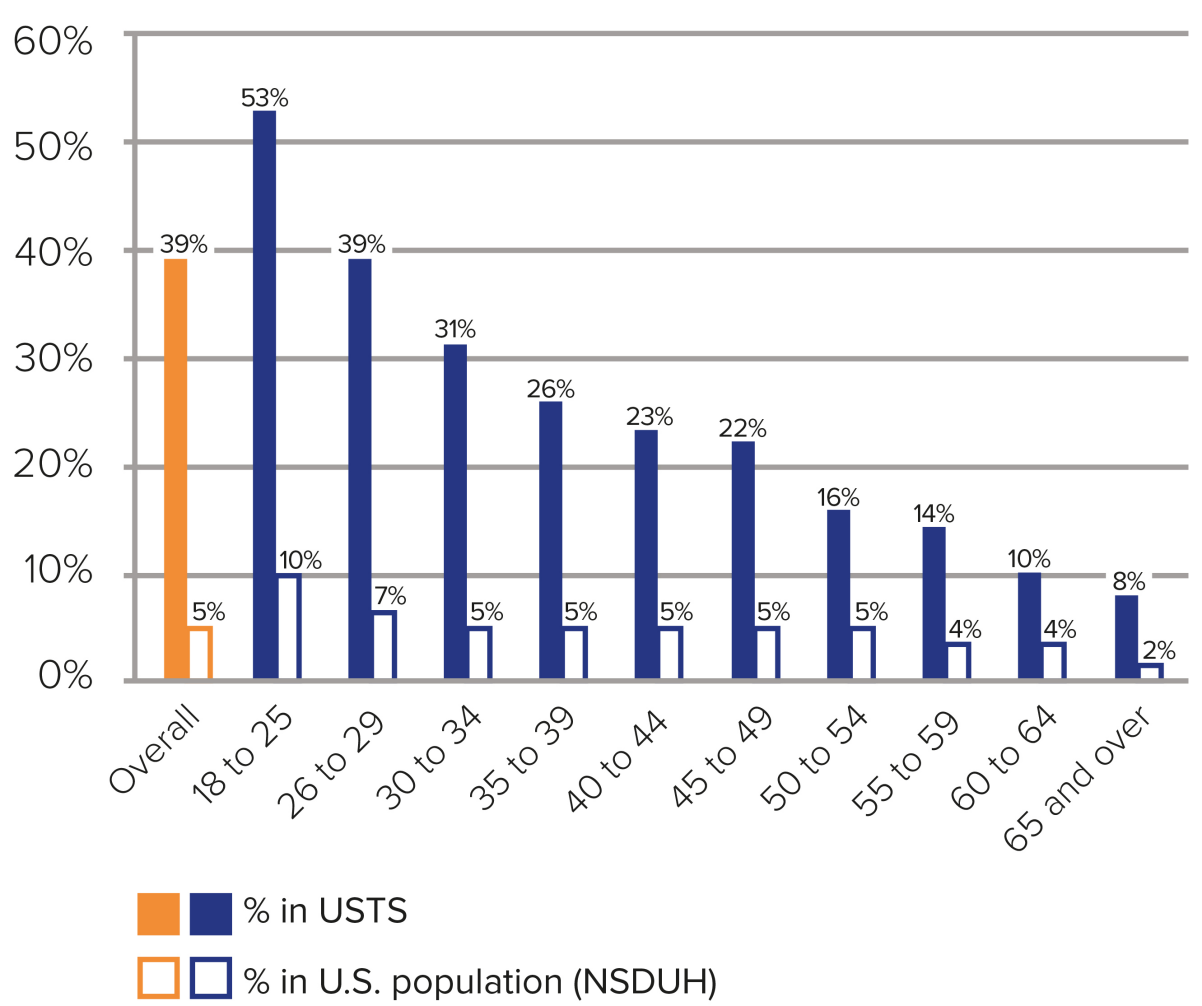
**Cross-sectionally reported experience of current serious psychological distress by transgender persons of different ages, as compared to the general US population**. Note that psychological distress is about 8 times higher in the transgender population, and that transgender youth experience particularly high levels of psychological distress. (Adapted from Fig. 7:22, in James *et al*., 2016 [27]. Washington, DC: National Center for Transgender Equality). USTS, United States Transgender Survey; NSDUH, National Survey on Drug Use and Health.

Serious psychological distress is experienced by transgender persons of all ages but as shown in Fig. 4 (Ref. [27]), it is more common in young TGNC people from different sociodemographic backgrounds [26, 28, 29]. While psychological distress among older TGNC persons results mainly from stigmatization, discrimination and ageism [30], the unmet mental health needs of younger subjects [10] has been ascribed (among others) to hesitancy in self-reporting because health systems are often non-inclusive and suffer from inadequate healthcare professional-patient communication [31, 32].

The “minority stress model” may explain the heightened vulnerability of TGNC people to psychiatric disorders [33]. The model encompasses both distal (e.g., stigma, prejudice, and discrimination imposed by others) and proximal (previous negative experiences, anticipation of stigma, or avoidance of discovery) stressors. According to this model, the stressful experiences can lead to symptoms of anxiety and depression [34, 35, 36]. Besides the usual life stressors, TGNC children and adults are additionally burdened by gender identity-related stressors such as alienation or rejection at home and social environments, bullying, verbal and physical abuse, sexual assault, stigma and discrimination [37]. As might be predicted, anxiety and depression are the most common psychiatric diagnoses in TGNC subjects, with prevalence rates as high as 84% and 68%, respectively, compared to 4–5% for both conditions in the general population [38, 39].

Emotional distress is a strong predictor of suicidal ideation, attempted suicide and suicide [40, 41]. Multiple factors make suicide notoriously hard to study and the accuracy of national suicide statistics is often questionable [42]. Nevertheless, if data from the USA are assumed to be representative of the global picture, it is alarming that 81% of transgender adults have thought about suicide, 42% have attempted suicide, and 56% have engaged in non-suicidal self-injury at some point in their lives [43].

Familial rejection (often associated with homelessness) is a major cause of attempted suicide among TGNC persons [44]. Typically, attempted suicides are more common among TGNC youths and adults compared to their cisgender peers. Even in societies with liberal attitudes such as the State of California (USA), depression and victimization are significant mediators of suicidal ideation and attempted suicide in TGNC youth [45, 46, 47]. A recent study of suicidal thoughts and behaviors (STB) in a “convenience sample” of first-year undergraduates at 71 universities across 18 countries reported that (i) TGNC students have a 140% greater risk for STB than their peers who do not identify as transgender, and (ii) that TGNC students have an increased risk of ideation persistence which transitions to attempted suicide [48]. In light of current events, it deserves pointing out that structural transphobia (exemplified by the introduction of restrictive laws on transgender and non-binary rights in 19 states of the USA) significantly increases the odds of anxiety and depression and the rate of attempted suicides among younger and older TGNC people [49, 50, 51]. While suicide and attempted suicide leave ugly mental scars left on family and friends (and the surviving victim), the burden on national budgets (e.g., due to lost productivity) are often less well appreciated [52].

Other stress-related psychiatric conditions found among TGNC persons include eating disorders, alcohol or substance use disorder, and self-injury [12, 52, 53, 54, 55]. Eating disorders in TGNC result largely from body objectification [56] and dissatisfaction with body image and stigma-based stressors [57, 58]. Drugs and alcohol misuse by TGNC youths seems to represent a coping mechanism for gender identity-related stress [59]. Non-suicidal self-injury is more prevalent among TGNC adolescents than adults, starting around 12 years of age, non-suicidal self-injury peaks around 15–16 years of age [60]. Other psychiatric conditions that transgender persons are more prone to than their cisgender peers are post-traumatic stress disorder (PTSD), bipolar disorder, borderline personality disorder, dissociative identity disorder (multiple personality disorder, obsessive compulsive disorder, schizophrenia or schizoaffective disorder) [61, 62, 63]. One study also reported that transgender persons may have higher likelihood of developing Alzheimer’s disease compared to their cisgender counterparts [64], an observation that aligns with evidence that suggests that stress can trigger (depression and) Alzheimer’s disease [65, 66]. Another important issue that deserves mention is that prevailing socio-legal conditions and attitudes that suppress honesty (“outing”) make psychiatric diagnosis and treatment even more challenging because gender dysphoria may be an underlying (but undisclosed/conceaked) confound in serious mental illness [32, 67] and other chronic illnesses [68].

Clearly, many TGNC individuals lack full mental health, which is defined as well-being that enables people to realize their abilities, handle stress, interact with others, work effectively, and contribute to their community. The WHO mantra “there is no health without mental health” emphasizes that mental health is an integral part of health and must be promoted, protected and restored. While promoting an inclusive global health agenda for transgender people [69, 70], the WHO cannot enforce policy; moreover, its influence is increasingly being challenged by geopolitical polarization and authoritarian regimes [71]. History and culture (a complex construct that includes multiple components such as knowledge, beliefs, morals, laws, and customs) [72] add to these challenges, but these may be mitigatable through cultural sensitivity and cultural competency in healthcare practice and medical education [73], as well as strategies that engage those who can shape public attitudes and bring about social change [74].

Improvements in the quality of life of TGNC people depend strongly on policy and legislative changes which are subject to cultural pressures. Bringing about such change therefore requires informed and persuasive dialogue, along with suggestions for feasible (culturally sensitive) solutions. Preparation for such dialogue might include a compilation of regional/national legislative and policy initiatives that promote gender diversity (e.g., https://rm.coe.int/combating-discrimination-on-grounds-of-sexual-orientation-and-gender-i/16809fb2b8) and regional- or country-specific insights into the barriers encountered by gender minorities, exemplified by one for Egypt [75].

Readers will have noticed that most of the above-cited data and literature originates from observations in Western Europe and North America; these regions share many socio-cultural characteristics which are very different from those of most people living in low-and-middle income countries (LMIC) or the so-called “Global South”. This unintentional bias reflects the scarcity of a strong body of literature on any aspect of health in transgender men and women [76, 77, 78, 79]. While poor human and material resources undoubtedly contributes to this situation, legal and stigmatizing factors with respect to non-heteronormativity sexual orientation and gender identity may also be significant contributory factors. But, more importantly, the paucity of robust geopolitical-specific studies impedes improvements in (mental) healthcare for TGNC persons [78, 79, 80].

## 2. Eight Actionable Ideas for Assuring Better Mental Health for Gender Minorities

### 2.1 Initiate Stratified Data Sets—More Focus on the “T” in LGBTQ+

Most available published data concerning non-heteronormative gender identities tends to lump transgenderism under the Lesbian, Gay, Bisexual, Transgender, Queer/Questioning, and others (LGBTQ+) umbrella, without adequate recognition of difference between gender identity and sexual orientation. This makes it difficult to build a clear picture of the specific mental and other health challenges and needs of TGNC people, and to examine and develop dedicated health programs for this small, but significant, sub-group of LGBTQ+ persons. Likewise, considering “The Missing ‘T’ in LGBT Research” may play a key role in developing policies that impact upon transgender persons [17].

### 2.2 Engage in Dialogue and Research

Disinformation finds fertile ground in the minds of people with insufficient background knowledge; as a result, they are more vulnerable to develop discriminatory behavior towards “Other” groups. Biomedical professionals have a responsibility to communicate with the public, community leaders and policy makers. They are qualified to explain the biological underpinnings of gender identity (and sexual orientation) in terms of genetics, developmental biology, physiology and brain structure [81, 82, 83]. They can also canvass for more research into the biological basis of gender identity; such research should be ethical [84] and ideally involve partnerships with TGNC persons at the planning, implementation, results interpretation and dissemination stages in the spirit of community-engaged research (CEnR) [85]. The recent insightful research on TGNC rights by political scientists [17] suggests the potential of intersectoral collaboration in influencing attitudes and policy.

Communication with lay audiences is a key pathway to change [86] and may account for the recent positive attitude of Europeans to gender diversity [87]. Destigmatization interventions must be sustained to be effective [88] and with the fact that “knowledge needs to be based on strong empirical science with terminology that is accurate, and not on assumptions or propaganda from special interest groups that seek social or political authority” in mind [89]. The number of sources of information on gender diversity and stigma-reduction strategies is growing, two examples of which may be found at https://www.act.gov.au/__data/assets/pdf_file/0009/2863593/Trans-and-Gender-Diverse-Awareness-Campaign-Stakeholder-Pack.pdf (Australia) and https://transgenderhealth.gov.mt/en/psychosocial-care/psychosocial-support/ (Malta).

### 2.3 Transform Early Education

Early education plays a vital role in shaping society and fostering inclusive communities. Therefore, introduction to the concept of gender diversity in schools is likely to be effective in promoting respect for those belonging to minorities, including gender diverse people [90]; it can also be expected to reduce bullying, victimization, actions of hate, and stigma, as well as self-injury and suicidality among TGNC youth. Reforms that aim to include gender diversity in school curricula are likely to generate uneasiness and objections; these can soothed by thoughtful discussions with parents and other stakeholders, as well as appropriate training for teachers. Here, it is worth observing that despite initial opposition to sex education in schools, time has shown the significant benefits of such programs, e.g., lower rates of teenage pregnancy and sexually transmitted infections (including HIV).

### 2.4 Build Human Connectedness

Self- or enforced social isolation and absence of trusted confidants which fuel minority stress can be reversed by encouraging family support, and trusted relationships through greater human inter-connectedness. All professionals involved in psychiatric care, as well as teachers and faith/spiritual leaders can actively contribute to these goals. In doing so, they will empower TGNC people by nurturing their positive identity development and resilience [91, 92], echoing the view that “focused interventions that primarily aim to increase self-esteem, social support and interpersonal functioning may prove to be useful in increasing the quality of life of transgender people” [38].

### 2.5 Develop Inclusive Mental Healthcare Services

By embracing the concept of universal healthcare (UHC) [93], the global health community has begun to redress the limited access to even the most basic healthcare in low- and middle-income countries (LMIC). As stated by Dr Tedros Ghebreyesus, Director-General of the WHO, “Mental health must be an integral part of UHC. Nobody should be denied access to mental healthcare because she or he is poor or lives in a remote place”. In fact, many higher-income countries also lack UHC because of the barriers faced by gender minorities when seeking any form of medical care [94].

Non-discriminatory and non-stigmatizing psychosocial and pharmacological interventions [95, 96] for gender diverse persons fulfils moral (human rights) obligations and makes economic sense [22]. Recent research reports a preference by TGNC patients to have their mental health care integrated within a primary multidisciplinary integrated (collaborative) care system [97]. Lastly, the potential of digital health care [98] should be better explored since they overcome the stigma barrier while reducing the costs of service provision.

### 2.6 Improve Professional Training

Transformation of TGNC care must come from within the medical profession which has sometimes, sadly, contributed to the discrimination against non-heteronormative sexual orientations and gender identities [99]. In this context, reiteration we would reiterate the Endocrine Society’s statement: “Medical Evidence, not politics, should inform decisions” (https://www.endocrine.org/news-and-advocacy/news-room/2024/statement-in-support-of-gender-affirming-care).

Reviews on the experiences and specific mental health needs of non-heteronormative persons highlight gaps in medical education [94, 100, 101, 102] and advocate for a focus on gender diversity in internal medicine, psychiatry and psychiatric nursing curricula; they emphasize the inclusion of modules on working with minority groups in a respectful and sensitive manner, independently of personal beliefs or gender/cultural stereotypes. Other useful instruments would be in-service training in the development of tailored medical, psychological and social interventions, orientated around The World Professional Association for Transgender Health (WPATH) Standards of Care [103], and creation of a list of reliable resources to facilitate knowledge updating. Additionally, medical staff could familiarizing professionals from other sectors (e.g., law enforcement—see [104]).

### 2.7 Create Centers for Gender Medicine

Gender affirmation through gender-affirming care (GAC) is known to alleviate psychological distress and psychiatric symptoms in TGNC youth [10, 105, 106]. However, these interventions are often either forbidden by law or surrounded by controversy that physicians must navigate skilfully. In this context, reiteration of the Endocrine Society’s statement “Medical Evidence, not politics, should inform decisions” (https://www.endocrine.org/news-and-advocacy/news-room/2024/statement-in-support-of-gender-affirming-care) is appropriate.

The establishment of interdisciplinary units or centers for gender medicine could prove a significant boost for ensuring the physical and mental health of TGNC. Such centers (sometimes integrated within larger centres focused on sexual and reproductive health) already exist, albeit in Western countries. These entities are best qualified and equipped to provide guidance, established pathways, or structured systems for GAC and to advise TGNC persons with/out the explicit desire for transition procedures. Importantly, they have the expertise to discuss the potential risks of transitioning procedures and to connect clients with psychosocial support during and after GAC [107]. Further, these centers can help promote healthcare for gender minorities in communities where this is lacking, e.g., Arab League States [108]. Lastly, in conjunction with learned societies such as The International Society for Gender Medicine, they can help build global platforms and networks to share expert knowledge on clinical management of TGNC persons and to develop best practices following guidelines and protocols that meet the highest ethical and clinical standards [109, 110].

### 2.8 Ban Unethical Practices

**Fig. 5.**
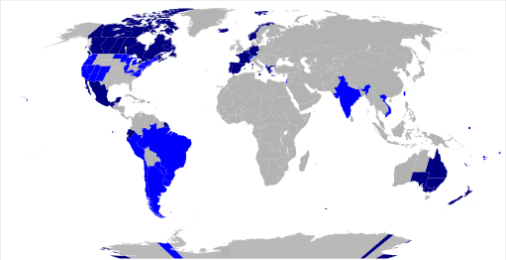
**Map of the world highlighting countries with different forms of bans on conversion therapy (2025)**. Dark blue indicates countries with a criminal ban on conversion therapy based on sexual orientation and gender identity; Light blue denotes countries where only medical professionals are prohibited from performing conversion therapy; Grey represents countries with no existing ban on conversion therapy. (Source: Stinger20, CC BY-SA 4.0 https://creativecommons.org/licenses/by-sa/4.0, via Wikimedia Commons.)

Gender Identity Conversion Efforts (GICE) or “conversion therapy” causes psychological harm that can persist throughout life; there is robust evidence for a causal association between GICE and increased risk of depression, TSD, and suicidality [111]. Nevertheless, GICE is still practiced in almost 170 countries (see Fig. 5 for countries with full or partial restrictions on the practice at the time of writing), despite condemnation by human rights and mental health organizations such as the Pan American Health Organization (PAHO) and World Psychiatric Association (WPA).

The interventions used in GICE include pseudo-scientific counselling sessions, exorcism, administration of “purifying” substances, food deprivation, threats of homelessness, physical violence, and/or “corrective” rape. Despite being based on pseudo-medical and often religious-based rituals, GICE practitioners claim to be able to alter, discourage, or suppress a person’s gender identity and to “correct” sexual orientation. Moreover, although gender identity does not have a pathological basis [7], GICE is commonly peddled as a “healing” against signs of TGNC status. Its proponents also allege that they can help individuals come to terms with the body they were born with, as well as identify the cause of a person’s expressed gender, be it “social contagion, trauma, mental illness, internalized homophobia, or flight from womanhood” [112]. Victims of this dangerous practice may be children, adolescents and young adults; young people whose guardians have low educational and socio-economic status, belong to marginalized ethno-racial groups, and/or have had a particular religious upbringing are disproportionately likely to be convinced about the powers of GICE [113]. The questionable practices used in GICE can only exacerbate the mental burden of being a gender diverse person—it must be stopped in all countries!

## 3. Conclusion

In summary, our message is twofold:

(1) By putting one or all of the above 8 recommendations into practice, we will be exercising our duty to defend the UN’s Chater on Human Rights.

(2) By presenting scientifically robust medical reasoning and evidence, we can hope to influence mindsets and bring about legislation that assures health and wellbeing for every person, regardless of their gender identity.
